# The impact of Hydroxyurea on the rates of Vaso–occlusive crises in patients with sickle cell disease in Saudi Arabia: a single–center study

**DOI:** 10.1186/s12873-022-00751-0

**Published:** 2022-11-29

**Authors:** Sahar Abdullah Alkhalifah, Miteb Alanazi, Majed Ali Almasaoud, Hazim Saeed Al-Malki, Faisal Mohammed Al-Murdhi, Mohammed Saad Al-hazzaa, Suliaman Musaed Al-Mufarrij, Mohammed Ali Albabtain, Abdulrahman Abdullah Alshiakh, Yazed AlRuthia

**Affiliations:** 1 Department of Clinical Pharmacy, College of Pharmacy, King Saud University, Riyadh, 11451 Saudi Arabia; 2 Department of Pharmacy, King Khalid University Hospital, P.O. Box 3145, Riyadh, 12372 Saudi Arabia; 3 College of Medicine, King Saud University, Riyadh, 11451 Saudi Arabia; 4 Pharmacoeconomics Research Unit, Department of Clinical Pharmacy, College of Pharmacy, King Saud University, Riyadh, 11451 Saudi Arabia

**Keywords:** Sickle cell disease, Hydroxyurea, Vaso-occlusive crisis

## Abstract

**Background:**

Vaso–occlusive crises (VOCs) are acute and common painful complication of sickle cell disease (SCD), and are the main reason behind the frequent emergency department visits among SCD patients. Hydroxyurea (HU) is an old and commonly used medication that demonstrated its effectiveness in reducing the risk of VOCs and the incidence of hospitalization. Although multiple studies have examined the impact of HU on the rates of VOCs, few have explored its effectiveness among SCD patients in Saudi Arabia.

**Methods:**

This was a single–center retrospective cohort study in which the electronic medical records of patients with SCD who have not had any previous exposure to HU prior to the initiation of HU treatment for ≥12 months were recruited. Paired t–test was conducted to examine the difference in the rates of VOCs, and levels of hemoglobin (Hgb), hematocrit (HCT), and platelet counts (PLT Ct) prior to the initiation of HU therapy and 12 months later. Multiple linear regression was conducted to examine whether age, gender, use of opioid analgesics, Hgb, HCT, and PLT Ct levels predict higher or lower rates of VOCs.

**Results:**

One hundred and fifty–six patients met the inclusion criteria and were included in the analysis. About 51% of the patients were males, and their mean age was 12.69 years. The mean HU dosage was 16.52 mg/kg/day, and the mean reduction in the rate of VOCs was 1.36 events per patient per year (95% CI [1.03–1.70], *p* < 0.0001) after the initiation of HU. Females were more likely to have greater reduction in the rates of VOCs in comparison to their male counterparts (β–estimate = 12.85, 95% CI [0.759–24.93], *p* = 0.0374).

**Conclusion:**

The use of HU results in a significant reduction in the rates of VOCs and emergency department visits. Future studies with robust research designs should be conducted to further examine the impact of HU on VOCs, hospitalization, and length of stay as well as compare HU to other newly approved medications for SCD, such as crizanlizumab.

## Background

Sickle cell disease (SCD) is an autosomal–recessive genetic illness that is caused by a mutation that replaces the sixth amino acid with valine resulting in the generation of irregular sickle–shaped erythrocyte that disrupt blood flow in small vessels [1, 2]. This vaso–occlusion leads to distal tissue ischemia, chronic hemolytic anemia, microvascular thrombosis, ischemic pain, tissue infarction, and poor quality of life [1]. Frequent sickling and continuing hemolytic anemia could lead to chronic organ damage and parenchymal injury and eventually result in substantial morbidity and early mortality [1, 2]. Although the phenotypic expression of SCD is well- known, environmental factors, such as infections, cold weather and air quality, genetic predisposition, and fetal hemoglobin levels have been shown to impact the manifestations of the disease [3]. In 2010, the number of newborns who have been diagnosed with SCD was estimated to be 305,800 globally, and is projected to increase by one–third to 404,200 patients in 2050 resulting in a total number of 14,242,000 newly diagnosed patients between 2010 and 2050 [4]. In Saudi Arabia, the prevalence of SCD varies significantly between different regions mainly due to the variable rates of consanguineous marriages [5]. The highest reported prevalence of SCD is in the Eastern region with a prevalence rate of 145 patients per 10,000 people, followed by the Southern region and Western region with prevalence rates of 24 patients per 10,000 people and 12 patients per 10,000 people, respectively,with two major phenotypes (e.g., the mild and severe phenotypes) [2, 6]. The mild phenotype is present among patients with the Arab/Indian haplotype and is characterized by elevated Hb F levels, whereas the Benin haplotype is more severe, and is most prevalent in the Western region [2].

Vaso–occlusion pain episodes are the hallmark of SCD accountable for acute systemic painful vaso–occlusive crisis (VOC) [7]. VOCs are unpredictable and often require immediate emergency care and hospitalization [8]. On the other hand, the chronic pain that accompanies SCD in many cases is not a mere continuation of VOC, but can be due to multiple reasons, such as, central sensitization, neuropathic pain, and avascular necrosis of bone at various joints (e.g., shoulders, hips, and ankles) [9]. Therefore, the quality of life of SCD patients is greatly impaired due to the recurrent painful episodes, organ failures, neurocognitive deficits, and early mortality [10]. According to an online questionnaire–based study that explored the impact of VOCs on health–related quality of life (HRQoL) among a sample of 303 adults with SCD in the United States, patients with frequent VOCs (e.g., ≥4 episodes) reported worse emotional and social functioning domains’ scores of the Adult Sickle Cell Quality of Life Measurement Information System (ASCQ-Me) [11]. In Saudi Arabia, the HRQoL of 629 adult SCD patients was assessed in two tertiary care hospitals in the Eastern and Southern regions using the Medical Outcomes 36-Item Short-Form Health Survey (SF–36) questionnaire and found that patients who were presented with fever, swelling, skin redness, and received blood transfusions had worse SF–36 domains’ scores [12].

There are several therapeutic agents that have been approved for the management of SCD, such as hydroxyurea, L-glutamine, crizanlizumab, and voxelotor [13]. Hydroxyurea (HU) is one of the oldest prescription drugs for the management of SCD that was approved by the United States Food and Drug Administration (USFDA) in 1998 and was until recently the only available therapy to ameliorate SCD severity [13]. HU has shown to decrease the rates of acute chest syndrome, acute pain episodes, hospitalization, and red blood cell (RBC) transfusions in both adults and children with SCD [14, 15].

In Saudi Arabia, the painful VOCs episodes among SCD patients are mostly managed by HU besides opioid (e.g., codeine, hydrocodone/paracetamol, hydrocodone/ibuprofen, oxycodone, morphine, hydromorphone, oxymorphone, methadone, diamorphine and fentanyl) and non–opioid analgesics (e.g., paracetamol, celecoxib, naproxen, ibuprofen, and ketorolac) [16]. However, the impact of HU on the rates of VOCs among SCD patients has only been examined in few studies and mostly among pediatric SCD patients despite the relatively high prevalence of SCD among certain communities in Saudi Arabia [17, 18]. Therefore, the aim of this study was to examine the impact of HU on the rates of VOCs and its impact on important hematological markers, such as Hgb, HCT, and PLT Ct among SCD patients in a large tertiary care referral center in Saudi Arabia.

## Methods

### Patient population

In Saudi Arabia, HU is most commonly prescribed for SCD patients to alleviate the pain and reduce the incidence of VOCs [19, 20]. VOC episode was defined as a hospitalization or care in emergency department or day treatment unit for sickle cell pain. Therefore, only patients with SCD with at least one incident of VOC, and who did not receive HU for ≥12 months prior to the initiation of HU treatment were included. Patients with no history of HU treatment, pregnant women, those with other unrelated types of pain (e.g., tooth pain, headache, trauma), and patients who have been retrospectively followed for less than 12 months after the initiation of HU treatment were excluded.

### Study design and data collection

This was a retrospective medical chart review in which the electronic medical records of SCD patients who have been seen in the hematology clinics at a university–affiliated tertiary care center in Riyadh, Saudi Arabia, on a regular basis were reviewed. Data collection sheet included age, gender, number of VOCs managed in the emergency department or required hospitalization in the 12 months prior to HU treatment initiation and 12 months post HU treatment, use of opioid analgesics, and hematological markers (e.g., hemoglobin (Hgb), hematocrit (HCT), reticulocyte count, and Platelet count per microliter (PLT Ct)). Patients with VOCs are often presented with intense pain in one or more body sites, such as legs, back, abdomen, knees, arms, and chest (e.g., acute chest syndrome) [2]. HU is administered as a single dose of 15 mg/kg/day, which can be titrated after 12 weeks by 5 mg/kg/day with a maximum dose of 35 mg/kg/day. Six medical and pharmacy interns were involved in the manual data retrieval and collection from the electronic medical records of 562 patients with SCD at the hospital. A graduate pharmacy student was involved in reviewing and verifying the collected data by randomly comparing the collected data with the patients’ electronic medical records. The data were retrieved from the electronic medical records between January 7, 2021, and November 18, 2021. It is hypothesized that SCD patients will have a significant reduction in the rates of VOCs (event/patient/year), an increase in the hemoglobin and hematocrit levels, and a decrease in the PLT Ct and reticulocyte counts [18, 21–23].

### Sample size calculation

The minimum sample size was estimated to be 147 patients based on α = 0.05, β = 0.95, power of 95%, and effect size of Cohen’s d = 0.3 for the difference in the mean number of VOCs at the baseline and 12 months later.

### Statistical analysis

Statistical analysis was conducted using SAS® version 9.4 (SAS institute, Cary, NC, USA). Descriptive statistics using means, standard deviations, frequencies, and percentages were conducted to present the baseline characteristics. Paired t–test was conducted to examine the mean difference between the baseline number of VOCs, Hgb, HCT, and PLT Ct, in the year that preceded the 12–month follow–up and 12 months later. In addition, multiple linear regression analysis was conducted to explore whether age, gender, hematological markers (e.g., baseline Hgb, HCT, and PLT CT levels), and opioid analgesics influence the rate of VOCs reduction.

## Results

Out of 562 electronic medical records for SCD patients that were reviewed, 406 patients did not have any observations prior to HU treatment resulting in 156 patients who met the inclusion criteria and were included in the analysis. Male and female patients were almost equally represented in the sample (50.64% vs. 49.36%). More than two–thirds of the patients (74.36%) were pediatric (e.g., < 18 years of age), and the mean age of the patients was almost 13 years. The mean number of VOCs in the 12 months that preceded the HU treatment was 2.13, and the mean levels of Hgb, HCT, reticulocyte count, and PLT Ct prior to follow–up are shown in Table 1. Opioid analgesics (e.g., tramadol, paracetamol–codeine) were prescribed among more than 75% the patients. Hydroxyurea mean dose was 16.52 ± 6.68 mg/kg per day. The mean number of VOCs, and levels of Hgb, HCT, PLT Ct, and reticulocyte count at baseline (e.g., in the 12 months that preceded HU treatment) and 12 months later are shown in Table 2. VOCs mean number was significantly reduced by more than 63% after the initiation of HU treatment (2.13 vs. 0.77, *p* < 0.0001). Similarly, the mean reticulocyte count (%) level significantly decreased after the initiation of HU treatment (10.31 vs. 0.722, *p* = 0.007). On the other hand, Hgb (g/L) (85 vs. 87.17, *p* = 0.353), HCT (%) (26.05 vs. 26.44, *p* = 0.496), and PLT Ct (383.13 vs. 394.76, *p* = 0.295) mean levels have increased after the HU treatment, however, this was not statistically significant.

**Table 1:** Patients’ baseline characteristics

Characteristic	*N* = 156
Gender	
Male, N (%)	79(50.64)
Female, N (%)	77(49.36)
Age (yrs.), mean ± SD	12.69 ± 6.83
Number of annual vaso–occlusive crises, mean ± SD	2.13 ± 2.94
Use of opioid analgesic, N (%)	118(75.64)
Hemoglobin (g/L), mean ± SD	85.62 ± 16.52
Hematocrit (%), mean ± SD	26.05 ± 6.30
Platelet count per microliter (mcL), mean ± SD	383.13 ± 192.27
Reticulocyte counts (%)	10.31 ± 12.87

**Table 2:** The mean number of vaso-occlusive crises (VOCs) and levels of hemoglobin (Hgb), hematocrit (HCT), platelet counts (PLT Ct), and reticulocyte count (%) before the initiation of hydroxyurea and 12 months later

Variable	Baseline, mean ± SD	12–Month follow-up, mean ± SD	Mean difference	95% Confidence limits for mean difference	***p***–value
VOCs	2.13 ± 2.94	0.77 ± 1.23	1.36 ± 2.11	1.03–1.70	< 0.0001
Hgb (g/L)	85.62 ± 16.52	87.17 ± 24.04	−1.83 ± 23.72	−2.06–5.72	0.3534
HCT (%)	26.05 ± 6.30	26.44 ± 7.51	−0.49 ± 8.66	−0.93 ± 1.91	0.4964
PLT Ct	383.13 ± 192.27	394.76 ± 202.96	−15.58 ± 178.5	−44.87–13.72	0.2951
Reticulocyte count (%)	10.31 ± 12.87	0.722 ± 2.27	10.07 ± 16.63	4.08–16.07	0.007

**Fig. 1:**
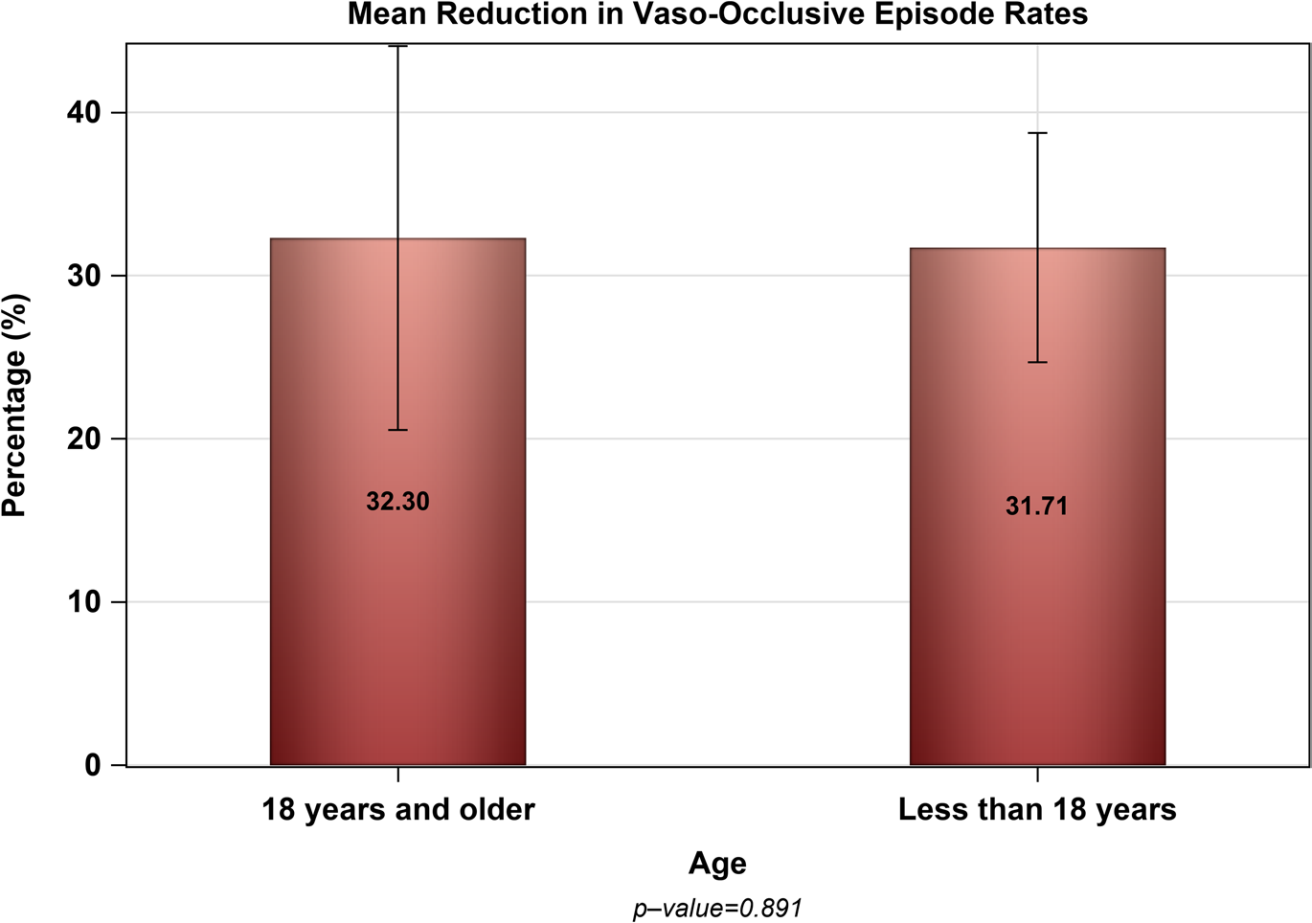
The mean percentage decrease in the rate of VOCs (event/patient/year) after the initiation of hydroxyurea among adults (≥18 yrs.) and pediatric population (< 18 yrs.)

**Fig. 2:**
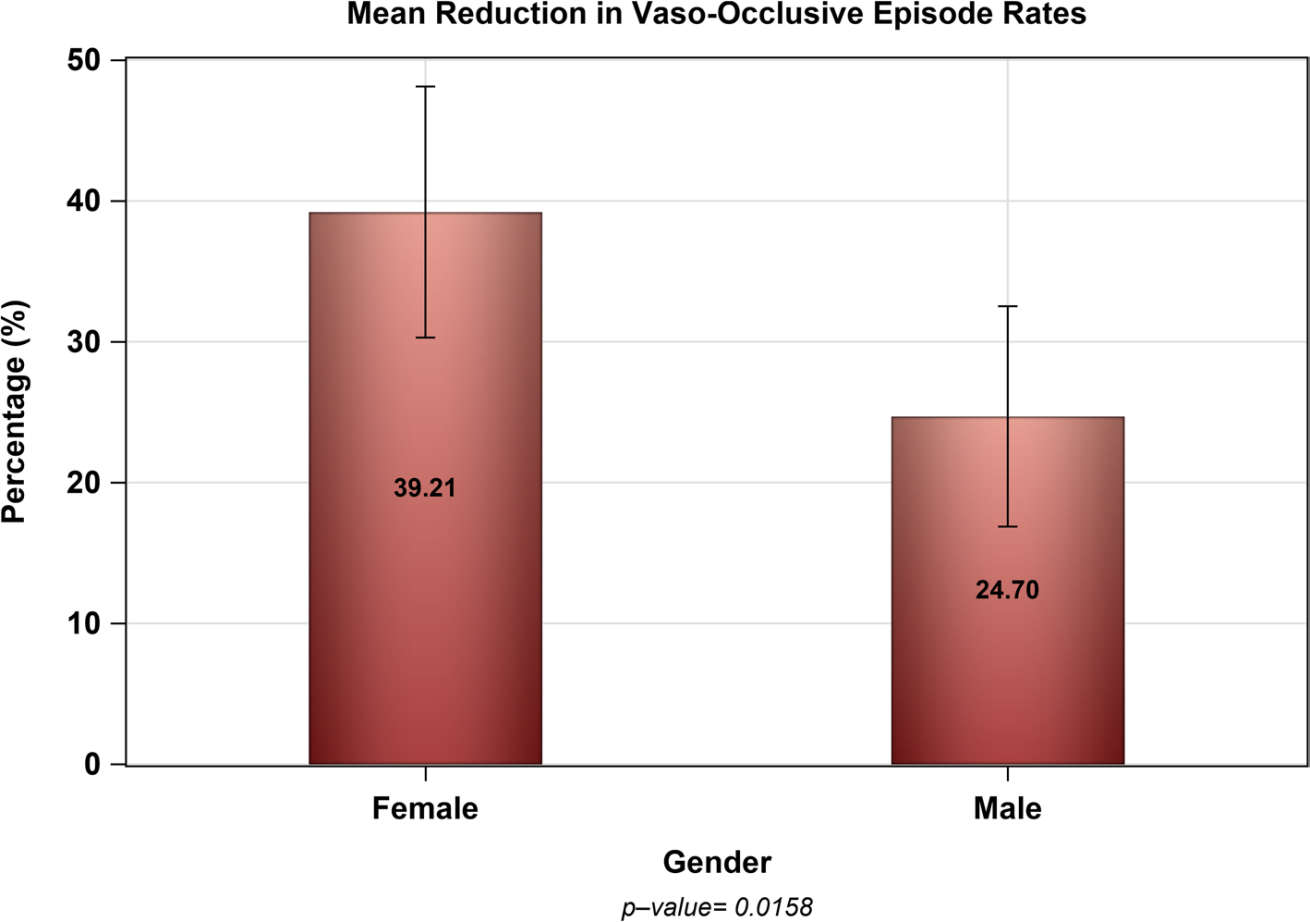
The mean percentage decrease in the rate of VOCs (event/patient/year) after the initiation of hydroxyurea among female and male patients

The mean reduction in the VOCs rate (event/patient/year) among patients aged 18 years and above was not significantly different than pediatric patients aged less than 18 years (32.30% vs. 31.71%, *p* = 0.891) as shown in Fig. 1. However, the mean reduction in the rate of VOCs (event/patient/year) among female patients was greater than their male counterparts (39.21% vs. 24.70%, *p* = 0.0158) as shown in Fig. 2. Furthermore, female gender was associated with greater reduction in the rate of VOCs (β = 12.85, 95% CI [0.759–24.93], *p* = 0.0374) controlling for age, baseline hematological markers (e.g., Hgb, HCT, PLT CT), and the use of opioid analgesics. However, baseline Hgb, HCT, and PLT CT as well as age and use of opioid analgesics were not associated with the rate of VOCs reduction as shown in Table 3.

**Table 3:** Multiple linear regression for the association between the rate of VOCs reduction over a 12-month period and age, gender, use of opioid analgesics, baseline Hgb, baseline HCT, and baseline PLT CT levels

Variable	β–estimate	95% Confidence limits	***p***–Value
Age	−0.365	−1.27– 0.547	0.430
Gender (female vs. male)	12.847	0.759–24.93	0.0374
Baseline Hgb	−0.135	−0.567–0.295	0.5351
Baseline HCT	−0.696	−1.818–0.424	0.2214
Baseline PLT CT	0.024	−0.0082–0.0562	0.1423
Use of opioid analgesics	11.865	−2.419–26.151	0.1028

## Discussion

The utilization of HU in the management of VOCs among SCD patients is common. However, little is known about its impact on VOCs rate among the Saudi SCD patient population [17, 18, 24]. Therefore, this study aimed to assess the impact of HU on the rate of VOCs among adult and pediatric SCD patients. Most of the studies that examined the clinical effectiveness of HU in the management of SCD have found it to be effective in reducing the rates of VOCs [14, 15, 18, 24], and was found to be effective in this study as well. However, the impact of HU on the rates of VOCs among SCD patients was not similar. For example, in a study that included 27 SCD patients aged 10 years and older who were recruited between 1994 and 1998 and were followed for 12 months in a single center in the Eastern region in Saudi Arabia, the use of HU resulted in a significant reduction in the rates of hospital admissions, VOCs, and hospital stays, and improvements in the hemoglobin and hemoglobin F levels [24]. Additionally, in another single center retrospective cohort study that was conducted among 82 pediatric SCD patients who were recruited between January 2012 and June 2017 in Riyadh, Saudi Arabia, the use of HU resulted in a significant reduction in both VOCs and hospital stay [18]. However, HU did not result in a significant reduction in the rates of VOCs among a sample of 102 pediatric SCD patients in a single center retrospective cohort study in the Western region of Saudi Arabia [17]. Therefore, the findings of the study are consistent with the results reported in other studies that were conducted in Saudi Arabia with similar SCD populations as well as with HU experience in other countries with SCD.

Female gender was found to be a significant predictor of lower rates of VOCs even after controlling for the use of opioid analgesics, hemoglobin, hematocrit, and platelet count at baseline, and age. This gender-related difference in the rate of VOCs reduction after the initiation of HU treatment might be related to the hormonal variations found in the two genders after puberty among adults and could be related to other unknown factors among pediatric SCD patient population as one single-center study that analyzed the clinical records of 39 pediatric patients in Italy has suggested [25]. In another study that examined the gender difference of sickle cell anemia (SCA) related complications among a sample of 248 patients in Nigeria, male patients were more likely to experience SCA related complications, such as sickle cell leg ulcer and avascular necrosis, than their female counterparts. However, the rates of painful crises were not significantly different between the two genders [26]. Moreover, in a single-center observational study at the Eastern region of Saudi Arabia, the rates of male SCD patients who were hospitalized due to painful crises post emergency room visits were more common among males than females [27]. On the other hand, the females reported higher rates of severe painful episodes, VOCs, and hospitalization in comparison to their male counterparts according to an observational study that analyzed data of 2124 patients from Sickle Cell Disease Implementation Consortium (SCDIC) registry [28]. The reticulocyte counts have been significantly decreased after the initiation of HU treatment, which is consistent with the preponderance of evidence [21, 29].

Although this study examined the impact of HU among both pediatric and adult SCD patients in Saudi Arabia with a relatively large sample size, it has multiple limitations that must be acknowledged. First, information bias cannot be excluded since this was a retrospective chart review with missing observations at the 12-month follow-up, such as the impact of HU on blood transfusions. Moreover, the impact of HU on the hospital length of stay, fetal hemoglobin, blood transfusions, white blood count (WBC), and mean corpuscular volume (MCV) was not examined due to the missing data in the electronic medical records. Additionally, this was a single center study which limits the generalizability of its results. Furthermore, the study did not include a control group to compare the rates of VOCs among patients who did not receive HU mainly due to the lack of data for patients who were not on HU. In addition, the patients’ SCD genotype was missing since the data were retrospectively retrieved from the electronic medical records and such information was not transferred to the patients’ electronic medical records and were rarely documented in their paper medical records. Finally, the patients’ mean HU dosages were relatively low in comparison to the ones reported in the literature which could explain the absence of any significant impact of HU on the hemoglobin levels [30].

## Conclusion

Hydroxyurea is an old and effective drug in the management of VOCs among SCD patients with seemingly favorable outcomes among female patients. Future studies with larger samples and more robust research designs should be conducted to further examine the impact of HU on VOCs, hospital admissions, length of stay, WBC, platelet, Hgb, Hgb F, and MCV. Also, the differences in SCD patients’ response to HU treatment based on their gender should be further examined in multicenter studies. Moreover, comparative effectiveness studies that compare the outcomes of more expensive and innovative medications for SCD, such as crizanlizumab, with HU should also be conducted to explore the incremental value of these new medications.

## Data Availability

The datasets used and/or analyzed are available from the corresponding author upon reasonable request.

## Abbreviations

VOCs: vaso–occlusive crises
SCD: sickle cell disease
HU: Hydroxyurea
Hgb: hemoglobin
HCT: hematocrit
PLT Ct: platelet counts
HRQoL: health–related quality of life
ASCQ-Me: Life Measurement Information System
SF–36: Medical Outcomes 36-Item Short-Form Health Survey
USFDA: United States Food and Drug Administration
RBCs: red blood cells
NAC: N–Acetylcysteine
SCA: sickle cell anemia
SCDIC: Sickle Cell Disease Implementation Consortium
WBC: white blood count
MCV: mean corpuscular volume
